# Molecular Mechanisms and Novel Therapeutics Targeting Ferroptosis in Gastric Cancer: A Literature Review

**DOI:** 10.7150/jca.119757

**Published:** 2025-10-20

**Authors:** Hsi-Lung Hsieh, Ming-Chin Yu, Hui-Ching Tseng, Yi-Hsuan Wu, Ming-Ming Tsai

**Affiliations:** 1 Graduate Institute of Health Industry Technology, Center for Drug Research and Development, College of Human Ecology, Chang Gung University of Science and Technology, Taoyuan 333, Taiwan.; 2 Department of Neurology, Chang Gung Memorial Hospital, Taoyuan 333, Taiwan.; 3 Department of Chemical Engineering, R&D Center of Biochemical Engineering Technology, Ming Chi University of Technology, New Taipei City 301, Taiwan.; 4 Department of General Surgery, New Taipei Municipal TuCheng Hospital, New Taipei 236, Taiwan.; 5 College of Medicine, Chang Gung University, Taoyuan 333, Taiwan.; 6 Department of General Surgery, Chang Gung Memorial Hospital, Taoyuan 333, Taiwan.; 7 Department of Nursing, Division of Basic Medical Sciences, Chang-Gung University of Science and Technology, Taoyuan 333, Taiwan.

**Keywords:** ferroptosis, gastric cancer, iron, biomarker, reactive oxygen species, antioxidant, redox, lipid peroxidation

## Abstract

Since the discovery of ferroptosis, which plays an important role in gastric cancer (GC), its activation has been crucial for developing tumor therapeutic strategies. Recently, ferroptosis activation has become a research hotspot for GC treatment approaches. Energy and metabolism dysfunctions involving lipids, amino acids, iron, sugars, and nucleotides caused by GC cells in a typical hypoxic microenvironment are important disease characteristics. However, the immune escape mechanism of GC cells limits the occurrence of programed cell death, a controllable form of which is ferroptosis. First, excessive reactive oxygen species production induces changes in intracellular iron ion levels, resulting in an imbalance in the antioxidant defense system. Finally, excessive accumulation of intracellular lipid peroxidation byproducts destroys cell membrane consistency and causes cell death. The promotion of ferroptosis in GC cells has been widely employed as a method for inhibiting tumor growth and chemotherapy resistance, which is helpful for developing anti-GC targeted treatments. Because GC cells are sensitive to ferroptosis-inducing agents, some traditional antitumor drugs (e.g., cisplatin) and Chinese herbal or natural medicines (e.g., artemisinin) exert anticancer effects by inducing this process. In this article, we summarize the basic molecular mechanisms underlying ferroptosis and the involved tumor markers, along with associated chemotherapy drugs and natural medicines. To activate ferroptosis in GC, new targeted drug therapies can be used within the clinical treatment field to kill GC cells and enhance tumor sensitivity to chemotherapy.

## Introduction

In 2003, Dr. *B.R. Stockwell* first discovered ferroptosis while screening for small-molecule drugs (e.g., erastin), which selectively kill tumor cells 1. In 2012, Dr. Dixon officially named it “ferroptosis” because the process involves iron dependency. Ferroptosis is classified as regulated cell death (RCD) by various intracellular small-molecule inducers and inhibitors 2.

**Figure 1:**
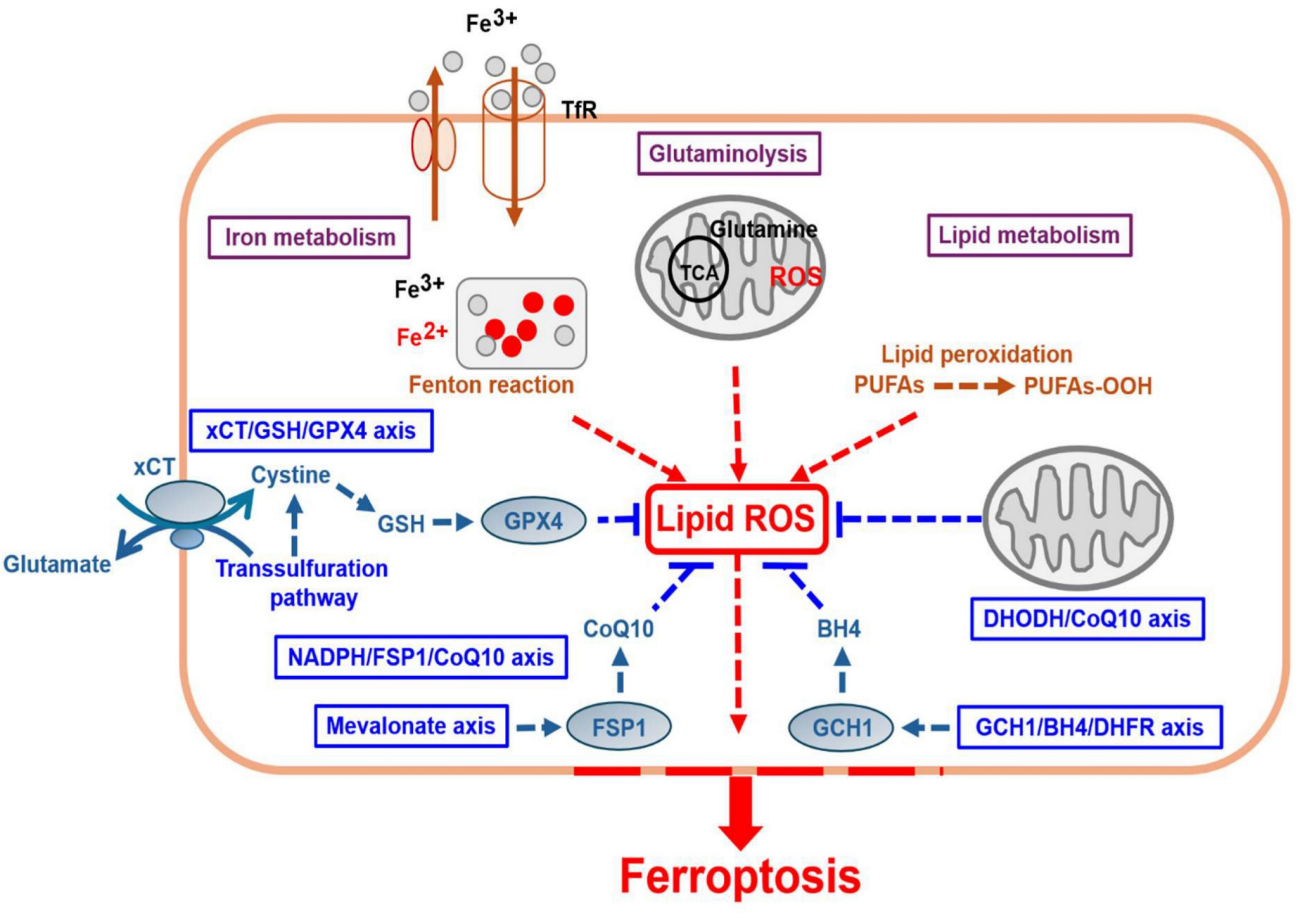
Ferroptosis-related proteins and molecular mechanisms.

Ferroptosis involves the activation of several energy and metabolic processes and substances, such as iron, amino acids, and polyunsaturated fatty acids, leading to intracellular redox imbalance and dysregulated generation and degradation of lipid reactive oxygen species (ROS) 3 (Fig. 1 and Table 1). Recent studies, including cancer research, have shown that ferroptosis can participate in gastric cancer (GC) progression, where pro- or anti-ferroptotic pathways are involved 4. This study discusses the mechanisms underlying ferroptosis and summarizes recent research findings to provide a reference for ferroptosis-based anti-GC strategies and contribute to the future development of new therapeutic approaches in clinical practice.

In this review, **PART I** delineates the molecular mechanisms underlying ferroptosis, with particular emphasis on the reprogramming of iron, lipid, and amino acid metabolism in GC cells. **PART II** identifies ferroptosis-related proteins as potential biomarkers, highlighting their clinical relevance in disease progression and poor prognosis. **PART III** explores the therapeutic potential of inducing ferroptosis through conventional chemotherapeutic agents, while **PART IV** extends this perspective to Chinese herbal medicines and natural compounds, underscoring their promise in overcoming chemoresistance. Finally, **PART V** addresses the remaining challenges of ferroptosis-based anti-GC therapy. Collectively, these sections underscore both the opportunities and the challenges that must be resolved to ensure the successful translation of ferroptosis-targeted strategies from experimental research into clinical application.

## PART I: The molecular mechanisms underlying ferroptosis activation in tumor cells (lipid peroxidation, intracellular iron accumulation, and loss of antioxidant defense)

### 1) Iron metabolism dysregulation (intracellular iron accumulation)

Iron metabolism is important for some cellular activities, including oxygen transport, respiration, and DNA synthesis 5. Iron is a key cofactor of cellular growth and metabolism. Ferric iron (Fe³⁺), which is carried by transferrin in circulation, is endocytosed into cells after binding to transferrin receptor 1 (TfR1) on an unaffected cell membrane 6. Within cells, ferric iron undergoes reduction to ferrous iron (Fe²⁺) and released into cytoplasm, and excessive iron is stored in ferritin (FE) 7. The latter is a universal intra- and extra-cellular protein that stores iron and is stored in ferroportin (FPN/iron transporter) 8-10. Free iron ions are released from ferritin degradation *via* NCOA4-dependent ferritinophagy 11. Iron primarily circulates through continuous redox cycling between Fe²⁺ and Fe³⁺ through the nonenzymatic Fenton reaction 12, leading to excess redox-active iron.

However, several tumor cells have dysregulated iron metabolism because of their rapid growth, with a higher overall intracellular iron concentration 13. Tumor cells increase iron uptake and storage, while reducing iron efflux. Moreover, iron can produce excessive ROS through the Fenton reaction, activating lipid peroxidation and making tumor cells more susceptible to ferroptosis. Therefore, various iron metabolism pathways and organelles (mitochondria or lysosomes) can modulate ferroptosis 14. Consequently, iron chelators 15 can inhibit ferroptosis, whereas iron-loaded nanoparticles 16 can induce ferroptosis in tumor cells.

### 2) Lipid metabolism (lipid ROS accumulation, redox imbalance, and lipid peroxidation of polyunsaturated fatty acids (PUFAs) leading to membrane damage)

In unaffected cells, glucose enters the cell to undergo glycolysis to produce pyruvate. Subsequently, pyruvate enters mitochondria and undergoes catalysis into tricarboxylic acid (TCA). The intermediates produced in this process contribute to lipid synthesis and the production of PUFAs. Iron-containing enzymes, such as arachidonate lipoxygenase (ALOX), mediate the lipid peroxidation pathway and promote the oxidation of free PUFAs. In cell membranes, PUFAs are transformed by acyl-coenzyme A synthetase long-chain family member 4 (ACSL4) into PUFA-CoA, which is then further catalyzed by lysophosphatidylcholine acyltransferase 3 (LPCAT3) into PL-PUFAs 17. However, in cancer cells, redox imbalance leads to excessive ROS production, further driving the peroxidation of specific membrane lipids. PL-PUFAs are highly susceptible to peroxidation by LOX, in which they are converted to phospholipid hydroperoxide (PLOOH). Excess lipid ROS, catalyzed by ALOX5, reacts with free radicals to form lipid peroxides, finally activating ferroptosis. Therefore, any factors that affect the expression and activity of ALOX5 can control the occurrence of ferroptosis 18. Furthermore, controlling the synthesis or degradation of PUFAs influences the cellular sensitivity to it. Moreover, the activity of iron-containing enzymes depends on iron availability, and the latter can also promote nonenzymatic lipid auto-oxidation through the Fenton reaction. If cells fail to effectively destroy peroxidized lipids, membrane damage occurs, leading to cell death 19. Iron chelators (iron scavengers) serve as effective inhibitors of lipid peroxidation and contribute to the occurrence of anti-ferroptosis 20.

### 3) Glutaminolysis

Glutaminolysis is a cellular metabolic process in which glutamine is converted into α-ketoglutarate (α-KG), involved in the TCA cycle to provide energy and metabolic intermediates. Recent studies have shown that glutaminolysis plays an essential role in regulating ferroptosis, primarily by influencing intracellular ROS, glutathione (GSH), and lipid peroxidation, thereby modulating cell survival and death. Glutaminolysis plays a dual role in regulating ferroptosis because it can promote lipid peroxidation progressing to ferroptosis and suppress the latter through antioxidant mechanisms. Regulating glutaminolysis can therefore be considered a new strategy for promoting ferroptosis, while providing new research directions for cancer treatment based on different tumor types and metabolic characteristics 21.

### 4) The classic xCT/GSH/GPX4 axis involves glutathione/glutathione peroxidase 4 metabolism (loss of antioxidant defense and redox imbalance)

In hypoxic microenvironments, tumor cells can generate energy using cystine in addition to glycolysis. Cystine/glutamate (Gln) is transported into cells *via* the Xc-(solute carrier family 3 member 2/SLC3A2) 22/xCT (solute carrier family 7 member 11/SLC7A11) 2 antiporter, and it is transformed into cysteine by the action of glutaminase (GLS), producing NADPH. Subsequently, GPX4 can promote the formation from GSH to glutathione disulfide (GSSG) and NADPH to NADP^+^ while directly converting endogenous phospholipid hydroperoxide (PLOOH) into harmless PL-OH 23. Therefore, both xCT (SLC7A11) and GPX4 exert crucial duplicated effects on antioxidant defense and ferroptosis inhibition by regulating intracellular GSH accumulation 24.

The second key component, the thioredoxin reductase 1 (TXNRD1)/thioredoxin (TXN)-dependent antioxidant system, mediates the reduction of cystine to cysteine through trans-sulfuration 25. In certain malignancies, the TXN-dependent cellular system is commonly activated, and inhibiting GSH and TXN pathways is a well-established method for effectively triggering cell death. In some cancer cells types, the trans-sulfuration pathway is continuously activated to increase cysteine levels 26. The enzymes involved in this pathway, including glycine N-methyltransferase (GNMT), S-adenosylhomocysteine hydrolase (SAHH), cystathionine β-synthase (CBS), and cystathionine gamma-lyase (CTH), sensitize cancer cells to ferroptosis and slow down tumor progression 27. Furthermore, GSH helps regulate the redox state within cancer cells, reducing their sensitivity to ferroptosis. Furthermore, some reports have indicated that amino acid transport (e.g., glutamine/Glu/SLC1A5/ASCT2/SLC38A1) 28, 29 and α-KG promote the TCA cycle, which increases ROS production and facilitates ferroptosis 30.

### 5) GPX4-independent regulatory pathwaysUnlike GPX4, which acts as a central suppressor of ferroptosis, the following four pathways regulate ferroptosis independently of GPX4

#### a) Ferroptosis suppressor protein (FSP1)/coenzyme Q10 (CoQ10/Ubiquinone)/nicotinamide adenine dinucleotide phosphate (NAD(P)H) axis

FSP1 is a CoQ oxidoreductase that protects cells from ferroptosis 31. Mechanistically, FSP1 possesses NADH-CoQ reductase activity, enabling the reduction of CoQ10 and NADPH to form CoQ10H2 (ubiquinol) and NADP^+^. This process directly reduces lipid radical formation or promotes the antioxidant regeneration of α-tocopherol (α-TOH/vitamin E), thereby inhibiting lipid peroxidation and ferroptosis. FSP1 exerts a concomitant inhibitory effect with GPX4 on ferroptosis 32, 33.

#### b) Mevalonate (MVA) axis

The MVA pathway is an integral part of ferroptosis regulation *via* CoQ10 synthesis, cholesterol metabolism, and protein translation. CoQ10 and FSP1 can hinder ferroptosis *via* antioxidant mechanisms, whereas cholesterol metabolism imbalance can promote ferroptosis by affecting GPX4 stability. Therefore, the regulation of the MVA pathway can be a new therapeutic strategy for cancer or other diseases 34.

#### c) Dihydroorotate dehydrogenase (DHODH) axis

DHODH plays a significant role in ferroptosis regulation by representing a mitochondrial antioxidant defense mechanism. DHODH is engaged in the *de novo* pyrimidine synthesis pathway in the inner mitochondrial membrane, where it facilitates the oxidation of dihydroorotate to orotate and reduction of ubiquinone (CoQ10) to ubiquinol (CoQ10H2). Recent studies have suggested that DHODH can suppress ferroptosis in the mitochondria by inhibiting lipid peroxidation by maintaining CoQ10 in this form. This function is similar to that of FSP1, which operates in cytosol. DHODH inhibition has been shown to promote ferroptosis by promoting ferroptosis-mediated cell death, particularly in cells with low GPX4 activity, making it a potential therapeutic target 35.

#### d) GTP cyclohydrolase (GCH1)/tetrahydrobiopterin (THB/BH4)/DHFR/NADPH axis

The GCH1/DHFR/BH4 activation pathway plays a crucial protective role in ferroptosis regulation by inhibiting intracellular antioxidant mechanisms and lipid peroxidation, thereby preventing cell death. In unaffected cells, GCH1/BH4 activates phospholipid mono-unsaturation. This pathway influences the composition of phospholipid fatty acids, *stimulat*ing the synthesis and incorporation of MUFAs. The latter are more resistant to lipid peroxidation than PUFAs by reducing cellular susceptibility to ferroptosis. Subsequently, BH4 inhibits lipid peroxidation as an antioxidant. BH4 is a potent antioxidant molecule that directly scavenges lipid peroxyl radicals, reducing lipid peroxidation accumulation and thereby inhibiting ferroptosis. Studies have reported that high GCH1 expression promotes BH4 synthesis, thus making cells more resistant to ferroptosis 36. DHFR maintains the BH4 cycle and enhances antioxidant capacity. DHFR is involved in the recycling of BH4, thereby ensuring stable intracellular BH4 levels and maintaining its antioxidant function. DHFR inhibition decreases BH4 levels, making cells more prone to lipid peroxidation and ferroptosis. GCH1 overexpression and ferroptosis resistance then occur in cancer cells 37. Studies have found that GCH1 upregulation in certain cancer cells increases BH4 levels, thus enhancing resistance to ferroptosis. This suggests that the GCH1/BH4 pathway can be a cancer treatment target. Inhibiting GCH1 or BH4 can increase ferroptosis sensitivity in tumor cells, thereby enhancing therapeutic efficacy. The GCH1/DHFR/BH4 activation pathway regulates ferroptosis through antioxidant mechanisms and phospholipid composition modifications 38. Activation of this pathway prevents lipid peroxidation and ferroptosis, whereas its inhibition can enhance ferroptosis sensitivity, providing new strategies for cancer treatment.

## PART II: Ferroptosis-related biomarkers in GC

Early diagnostics of GC is complicated, causing high recurrence and mortality rates, along with low therapeutic response rates. Therefore, determining biomarkers that assist in GC diagnostics and targeted treatment is particularly important. Currently, biomarkers associated with ferroptosis (Table 2) are associated with the detection of GC.

### 1. High expression of TfR1

Because of the increased iron demand of GC cells, the ability of GC cells to acquire iron depends on the number of TfR1s. Although tumors often upregulate TfR1 to acquire iron, they also upregulate ferritin to sequester iron and reduce free iron levels. Furthermore, their activation can promote the storage of free iron, thereby inducing ferroptosis 42. Dysregulation or dysfunction of the iron transport system is considered an important factor for triggering ferroptosis.

### 2. Low expression of lipoxygenases 15 (LOX15)

Recently, Zhang *et al.* 43 found that exosomes containing miR-522 released by cancer-associated fibroblasts in the GC tumor microenvironment inhibit ferroptosis in GC cells by acting on LOX15, thereby promoting tumor growth and reducing GC sensitivity to chemotherapy with cisplatin and paclitaxel.

### 3. Low expression of cysteine dioxygenase 1 (CDO1)

CDO1 promotes the oxidation of cysteine. Hao *et al.* 44 found that CDO1 affects ferroptosis by limiting cysteine synthesis for that of GSH. The low CDO1 expression in GC cells may lead to the upregulation of GPX4 levels in GC cells, thereby inhibiting ferroptosis.

### 4. High expression of perilipin 2 (PLIN2)

PLIN2 is a protein associated with intracellular lipid metabolism and can act as an oncogene. PLIN2 participates in the regulation of the ferroptosis pathway in GC cells through genes, such as PRDM11 and IPO7. Its overexpression leads to the formation of a relatively hypoxic intracellular environment, which in turn inhibits ferroptosis and facilitates GC progression 45.

### 5. High expression of stearoyl-CoA desaturase 1 (SCD1)

SCD1 is a key enzyme in cellular fatty acid conversion and is a known oncogene in GC. SCD1 is highly expressed in GC tissues and can promote the proliferation and migration capabilities of GC cells. Studies have reported that in GC, SCD1 elevates the expression levels of ferroptosis inhibitors, such as GPX4 and SLC7A11, thereby increasing the resistance of GC cells to ferroptosis. SCD1 suppression significantly activates ferroptosis and hinders the growth of GC cells, revealing the mechanism of action of the oncogene SCD1 from the perspective of ferroptosis 46.

### 6. High expression of glutathione peroxidase 4 (GPX4)

GPX4 is a crucial inhibitor of ferroptosis and is commonly used as a biomarker for antioxidant systems in various disease models. The data analysis by Wang *et al.* 47 on the expression of GPX4 in GC suggests that the transcriptional level (mRNA) of GPX4 is significantly higher in GC tissues and indicates poor prognosis.

### 7. High expression of solute carrier family 7 member 11 (SLC7A11/Xc)

SLC7A11 is a key gene that inhibits ferroptosis. It plays an important role in preserving redox homeostasis. Although SLC7A11 primarily exerts a tumor-promoting effect, its overexpression also exposes lethal vulnerability in cancer cells, making them more sensitive to glucose or glutamine starvation 48.

### 8. Low expression of FSP1

Iron-dependent oxidative damage to cell membrane is a characteristic of ferroptosis, and this can be counteracted by FSP1 49.

### 9. High expression of activated transcription factor 2 (ATF2)

Recent studies have shown that ATF2 is noticeably upregulated in GC tissues, which indicates poorer clinical prognosis. In particular, ATF2 suppresses sorafenib-provoked ferroptosis, thereby reducing a drug's anticancer efficacy. ATF2 negatively regulates ferroptosis by upregulating heat shock protein 110 (HSPH1/HSPH105/HSPH110), which stabilizes SLC7A11 and prevents ferroptotic cell death 50.

### 10. High expression of Aurora kinase A (AURKA)

AURKA was found to be upregulated in GC and is a poor prognostic factor. Loss of ARID1A expression is associated with poor survival rates in GC. Downregulation of miR-4715-3p leads to AURKA overexpression, maintaining GPX4 levels and suppressing ferroptosis; restoring miR-4715-3p or inhibiting AURKA downregulates GPX4 and induces GC cell death 51.

### 11. High expression of B-cell receptor-associated protein 31 (BAP31)

BAP31 is involved in tumor progression, whereas the roles and action of BAP31 in GC remain widely unclear. We detected BAP31 upregulation in GC, which indicates poor survival rates. BAP31 knockdown suppressed cell growth and promoted G1/S arrest. Moreover, BAP31 attenuation elevated the lipid peroxidation level of a membrane and promoted cellular ferroptosis. Mechanistically, BAP31 modulated cell proliferation and ferroptosis by directly binding to VDAC1 with further oligomerization and polyubiquitination. HNF4A was linked to BAP31 as a promoter and promoted transcription. Furthermore, the knockdown of BAP31 tended to make GC cells sensitive to 5-fluoruracil (5-FU) and the ferroptosis inducer, erastin, *in vivo* and *in vitro* 52. Our review suggests that BAP31 can be a predictive factor for GC; furthermore, we recommend the elaboration of its treatment strategy.

### 12. Low expression of E-cadherin (CDH1)

In diffuse-type GC (DGC) cells, loss-of-function mutations in E-cadherin lead to higher sensitivity to nonapoptotic, iron-dependent types of cell death, such as ferroptosis. Homotypic interactions between its molecules and cells suppress ferroptosis by activating the Hippo pathway. Furthermore, single-nucleotide mutations detected in DGC abrogate the homophilic binding ability of the molecular marker, thereby reducing its capability to inhibit ferroptosis in cell culture and xenograft models. Importantly, although its loss in malignant cells is a key factor for epithelial-mesenchymal transition (EMT) and subsequent metastases, we found that circulating DGC cells lacking E-cadherin expression decreased metastatic potential due to higher susceptibility to ferroptosis. In summary, this study suggests that E-cadherin is a biomarker that enables prognosis determination for the primary tumor and circulating DGC cell sensitivity to ferroptosis, serving as a guide for future therapeutic schemes aimed at promoting ferroptosis 53.

### 13. High expression of CDGSH iron sulfur domain 1 (CISD1)

CISD1/mitoNEET and CISD2 hinder ferroptosis by decreasing mitochondrial iron uptake. CISD1 is a member of the CDGSH domain-containing family and can participate in the modulation of mitochondrial oxidative capacity. Overexpression of CISD1 can reduce mitochondrial iron accumulation and increase resistance to ferroptosis 54.

### 14. High expression of doublecortin-like kinase 3 (DCLK3)

DCLK3 is associated with poor prognoses in GC and modulates ferroptosis and mitochondria functioning *in vitro* and *in vivo*. DCLK3 was found to be upregulated in GC, and high DCLK3 expression is significantly correlated with low survival rates in GC. Here, DCLK3 knockdown lowered GC cell proliferation, provoked ferroptotic cell death, and increased oxidative stress levels. Logistic regression analysis revealed that TCF4 was an independent prognostic factor for GC. In particular, DCLK3 facilitated higher TCF4 expression and that of TCF4 downstream target genes (c-Myc and cyclin D1). Furthermore, DCLK3 overexpression promoted GC cell proliferation while decreasing ferroptotic cell death and oxidative stress. The underlying mechanism may involve the upregulation of TCF4, c-Myc, and cyclin D1. Our study suggests that DCLK3 regulates the levels of iron and reactive oxygen and is engaged in the TCF4 pathway, thereby facilitating GC cell growth. This indicates that DCLK3 can serve as a prognostic marker and therapeutic target in GC 55.

### 15. Low expression of glutaminase 2 (GLS2)

GLS2 activates ferroptosis by converting glutamate, which is a known suppressor of GC cell growth, which thus indicates poor prognosis in GC. Studies have reported the significant role of GLS2 as a tumor suppressor in malignancies, such as GC 56.

### 16. High expression of microsomal glutathione S-Transferase 1 (MGST1)

MGST1 expression indicates poor prognosis, activating the Wnt/β-catenin pathway by modulating AKT and hindering ferroptosis in GC. MGST1 expression was higher in GC and exhibited a correlation with low overall survival rates 57.

### 17. High expression of nuclear factor-erythroid 2 (NRF2)

High expression of NRf2, a transcription factor, may have a dual impact on the prognosis of patients with gastric adenocarcinoma. Ferroptosis may participate in tumor formation and affect the resistance of particular cancers, including GC, to medications 58.

### 18. Low expression of polycyanin B (PB)

A new GPX4 inhibitor called PB has been demonstrated to inhibit the expression of GPX4 in GC cells, leading to their death. Polyphyllin B hinders tumor growth by regulating iron metabolism and promoting ferroptosis 59.

### 19. High expression of SLC1A5, ANGPTL4, and CGAS (ferroptosis-related genes/FRG signature)

A novel FRG signature that could make a prognosis and show tumor immune microenvironment was constructed. In our study, a novel ferroptosis-related signature was created to predict outcomes in patients with GC. The ferroptosis-related signature reflected ferroptosis in GC, and with univariate Cox regression analysis, survival genes were evaluated to elaborate a prognostic model involving three genes (i.e., SLC1A5, ANGPTL4, and CGAS), which could predict survival rates in GC 60.

### 20. High expression of signal transducer activator of transcription 3 (STAT3)

STAT3 plays a critical role in oncogenic signaling and is activated in many types of human cancer. As an oncogene, STAT3 is a major signal transduction pathway involved in multiple cellular processes (e.g., proliferation and survival) 61. Additionally, STAT3 is a key negative regulator of ferroptosis in GC. Inhibition of STAT3 downregulates antioxidant and iron homeostasis genes (such as SLC7A11, GPX4, and FTH1), induces lipid peroxidation and Fe²⁺ accumulation, and reverses 5-FU chemoresistance 62.

### 21. High expression of zinc finger protein 36 homolog (ZFP36)

Genetic and pharmacological inhibition of STAT3 triggers ferroptosis by the transcriptional regulation of GPX4, SLC7A11, and FTH1 in GC 63.

In summary, if there is overexpression of amino acid antioxidants, or the low expression of lipid peroxidation-promoting enzymes, the effect is considered to be the same: the inhibition of ferroptosis. These findings reflect specific molecular targets and theoretical references for those of redox homeostasis to prevent and treat ferroptosis-related diseases.

## PART III: Ferroptosis-related chemotherapeutic drugs for GC

Recently, the relationship between chemotherapy action and ferroptosis has been considered an important topic in cancer research. Certain chemotherapeutic drugs can enhance anticancer effects by inducing lipid peroxidation, affecting GSH metabolism, or inhibiting antioxidant systems to promote ferroptosis. Currently, those commonly used in GC treatment are mainly represented by platinum-based fluoropyrimidines and taxanes. However, multidrug resistance in patients with GC remains a major issue in chemotherapy. Cancer cells may undergo both apoptosis and ferroptosis simultaneously. As ferroptosis represents a novel mode of RCD that is completely not associated with apoptosis, it provides a new approach for overcoming chemotherapy drug resistance. Targeting ferroptosis to counteract chemotherapy resistance and integrating it within other therapeutic strategies represents a promising method for improving cancer treatment outcomes. Table 3 presents the chemotherapy drugs associated with ferroptosis in GC.

### 1. Platinum-based chemotherapy (cisplatin and oxaliplatin)

Studies have demonstrated that ATF3 inhibits the Nrf2/Keap1/xCT signaling pathway. Therefore, targeting this pathway with the aim to induce ferroptosis leads to increased intracellular ROS levels, depletion of intracellular GSH, or downregulation of GPX4, thus promoting lipid peroxidation accumulation. This helps overcome cisplatin resistance because GC cells are sensitized to cisplatin through ferroptosis 64, 65. Another chemotherapy drug, oxaliplatin, may act on ferroptosis through the miR-181a-5p/SIRT1 pathway 66, 67.

### 2. Paclitaxel-based (sorafenib)

Sorafenib is a tyrosine kinase inhibitor that promotes ferroptosis through the SLC7A11-related pathway mediated by the downstream target gene HSPH1 of ATF2. This further supports the potential of chemotherapy combined with ferroptosis-targeted therapeutic strategies. Furthermore, silencing sirtuin 6 (SIRT6) inactivates the Keap1/Nrf2 signaling pathway, which helps overcome sorafenib resistance 50.

### 3. 5-FU-based (capecitabine and 5-FU)

ADP-ribosylation factor 6 (ARF6) regulates erastin-induced ferroptosis and lipid peroxidation. Inhibiting ARF6 can reduce capecitabine resistance in GC cells. Another chemotherapy drug, 5-FU, promotes ROS-mediated ferroptosis. Drug resistance in cancer cells is a major cause of treatment failure. Numerous studies have indicated that drug resistance mechanisms in GC are often associated with dysregulated iron metabolism, antioxidant pathways, and prolonged exposure to ROS at excessive levels, along with the accumulation of lipid peroxides 68. Subsequently, ferroptosis resistance is considered a key molecular mechanism contributing to tumor drug resistance 69.

## PART IV: Ferroptosis-related Chinese herbal or natural medicines for GC

Recent studies have found that traditional Chinese medicines (TCMs) and their natural byproducts are involved in ferroptosis pathways in tumor cells and can regulate ferroptosis in GC cells, ultimately achieving therapeutic effects. If TCM-derived compounds-induced ferroptosis is prevented by ferrostatin-1, liproxstatin-1, or deferoxamine (an iron chelator) but not by pan-caspase inhibitors (such as Z-VAD-FMK), and is associated with lipid peroxidation, GPX4 inhibition, iron dependence, and distinct mitochondrial morphological alterations, it provides direct evidence of ferroptosis and represents the standard for distinguishing ferroptosis from apoptosis, pyroptosis and necroptosis 2, 70. Herbal medicine has unique advantages in cancer treatment, including multiple targets, fewer side effects, strong efficacy, lower economic burden, structural stability, high safety, low cost, and easy accessibility. In addition to prolonging patients' survival, it also improves their quality of life, and its clinical efficacy has been increasingly recognized by both medical professionals and patients. The pathways associated with ferroptosis in GC cells and the mechanisms through which herbal medicine exerts its effects, are worth further exploration to provide theoretical support for clinical GC treatment.

Considering recent research on the relationship between TCM and ferroptosis in GC cells, this review focuses on how herbal medicine regulates this process in GC treatment. It aims at providing new insights into therapeutic strategies for GC. Currently, drug therapy remains the primary approach in cancer treatment. However, acquired drug resistance continues to be a major challenge in clinical practice. Combination therapy can enhance drug efficacy, reduce side effects, and slow or prevent the development of resistance. Studies have shown that certain Chinese herbal medicines and their active components possess both anticancer and chemosensitizing effects, significantly increasing the sensitivity of tumor cells to chemotherapy and molecular-targeted drugs, thus improving therapeutic outcomes and reducing adverse reactions. Table 4 summarizes the mechanisms and applications of Chinese herbal medicines and their natural byproducts involved in targeting ferroptosis in cancer treatment, offering new insights into cancer therapy and overcoming drug resistance.

1. Amentoflavone (AF), a natural bioflavonoid compound, suppresses GC cell proliferation and promotes ferroptotic cell death. In particular, AF upregulates miR-496**,** which directly targets and downregulates transcription factor 2 (ATF2)**.** ATF2 inhibits ferroptosis; thus, its suppression by miR-496 facilitates ferroptotic processes in GC cells. miR-496 inhibition reverses AF's effects, highlighting the critical role of the miR-496/ATF2 axis in mediating ferroptosis in GC (AGS and HGC-27) 71.

**2. Metformin,** a widely used antidiabetic medication, induces ferroptosis in GC cells through the disruption of the STAT1-PRMT1 axis in HGC-27 and AGS cells, inhibition of the Nrf2 pathway, activation of p53, and enhancement of chemosensitivity 72, 73.

**3. Baicalein,** a flavonoid compound derived from the TCM herb *Scutellaria baicalensis*, promotes ferroptosis in GC cells through multiple mechanisms, including the disruption of iron homeostasis, inhibition of antioxidant defense, activation of the p53 pathway, and enhancement of chemotherapy sensitivity. Baicalein regulated p53-mediated SLC7A11 downregulation, leading to lipid peroxidation accumulation. It also induced ferroptosis by reducing MDA levels and PTGS2 expression while increasing SGC-7901 cell survival 74.

**4. Artemisinin**, a sesquiterpene lactone derived from *Artemisia annua*, is renowned for its antimalarial properties. Recent studies have unveiled its potential in inducing ferroptosis in various cancer cells, including those in GC, by regulating iron metabolism, inducing oxidative stress, and having selective cytotoxicity 75.

**5.** The root of Chinese kiwifruit ***Actinidia chinensis Planch* (*ACP*)** is a well-known TCM that has been approved for use as an adjuvant treatment for various diseases, including leukemia and lung, gastric, colon, and liver cancers, apart from promoting apoptosis in cancer cells. *ACP* extract is rich in ursolic acid (UA) and oleanolic acid (OA). Gao *et al.* 76 found that the expression of GPX4 and SLC7A11 was significantly inhibited in HGC-27 cells treated with ACP, ultimately accelerating the occurrence of ferroptosis. These outcomes suggest that targeting ferroptosis can be a novel approach for treating GC. Interestingly, GC cells of different histological types may exhibit varying degrees of sensitivity to ferroptosis-targeting drugs, with mesenchymal GC stem cells expressing it more than intestinal-type GC cells 77. In contrast, this may be attributable to the former containing more lipid-associated with ferroptosis; it is related to the increased activity of NRF2-dependent antioxidant pathways and SLC40A1 in intestinal-type GC cells.

**6. *Yiqi Jiedu Huayu decoction* (YJHD)** provokes ferroptosis in cisplatin-resistant GC cells by inhibiting the AKT/GSK3β/NRF2 signaling pathway, leading to decreased GPX4 expression and higher oxidative stress. This mechanism contributes to overcoming chemoresistance and highlights the potential of YJHD as an adjunctive therapy in GC treatment 78, 79.

**7. Ophiopogonin B (OP-B),** a natural compound extracted from *Ophiopogon japonicus*, promotes ferroptosis in GC cells through specific molecular mechanisms. Ophiopogonin B induces ferroptosis in GC cells by suppressing the GPX4/xCT antioxidant system, resulting in elevated lipid peroxidation and iron accumulation. This mechanism highlights OP-B's potential as a therapeutic agent targeting ferroptosis in GC 80.

**8. Jiyuan oridonin A (JDA)** is a natural compound obtained from the TCM herb *Jiyuan Rabdosia rubescens*. Its derivative, **compound a2 (a2)**, provokes ferroptosis in GC cells through several mechanisms, including downregulation of GPX4, iron accumulation *via* autophagy, with efficacy superior to that of 5-FU and favorable pharmacokinetics 81.

**9. Tanshinone IIA (Tan IIA),** an extract from the TCM *Salvia miltiorrhiza* (*Danshen*), is widely used in clinical settings to treat cardiovascular diseases. Furthermore, it has shown antitumor activity in various human cancer cell lines, including liver, gastric, colon, and lung cancers. Recently, Guan *et al.* 82 discovered that ferroptosis is a mechanism through which Tan IIA exerts its antitumor effects in GC. In BGC-823 and NCI-H87 cells, Tan IIA promotes lipid peroxidation and downregulates the expression of SLC7A11, thereby inducing ferroptosis and inhibiting GC cell proliferation. The ferroptosis inhibitor Fer-1 attenuates its anticancer effects. Furthermore, Tan IIA can upregulate wild-type p53 expression, suppress SLC7A11 expression in GC cells, and reduce intracellular cysteine and GSH levels, ultimately leading to ferroptosis 82. Furthermore, Tan IIA promotes GCSC stemness markers (e.g., *Octamer binding transcription factor OCT3/4* (OCT3/4) and Aldehyde dehydrogenase 1 family member A1 (ALDH1A1)), which reduces chemoresistance and enhances chemotherapy sensitivity 83.

In summary, several TCMs and their natural byproducts contribute to ferroptosis in GC cells. These herbal medicines have shown good efficacy in treating GC corresponding to their indications. They primarily induce ferroptosis by downregulating GPX4 and SLC7A11 (xCT) and upregulating p53, promoting lipid peroxidation and iron ion accumulation. This provides new research directions and potential applications for GC treatment. Therefore, these herbal medicines may inhibit the proliferation of GC cells by modulating ferroptosis.

## Part V: Further challenges of anti-GC therapy using ferroptosis

Recent studies on ferroptosis have enhanced our comprehension of its molecular mechanisms. In particular, the selective induction of ferroptosis in GC cells has become a prominent area, providing a new approach for cancer treatment. In this review, we elaborated on the mechanism underlying ferroptosis, including iron, lipids, amino acids, material metabolism, and nucleotides, whereas biomarker research on ferroptosis is of vital importance in the progression, diagnostics, treatment, and prognosis of GC.

The highly active metabolic capacity of GC cells makes them particularly susceptible to ferroptosis; therefore, treatment targeting the ferroptosis pathway and involving various chemotherapeutic drugs shows therapeutic potential to overcome drug resistance. However, many limitations should be acknowledged. Many GC cells can escape ferroptosis by activating or remodeling stress pathways, thus limiting the antitumor effects of ferroptosis inducers. With the continuous development of research, the regulatory mechanism underlying ferroptosis has been increasingly investigated. Researchers are seeking factors that make cells sensitive or resistant to ferroptosis. First, the molecular mechanism and metabolic basis of GC cells' escape from ferroptosis have not yet been elucidated. Second, the clinical application of ferroptosis in treating GC still requires a large amount of data and extensive experiments. More importantly, the complex interactions between ferroptosis and the tumor microenvironment causing tumor heterogeneity in GC can also have an impact.

Many TCMs and natural products have shown synergistic effects with conventional chemotherapy or targeted drugs in various types of GC, which can reduce systemic toxicity, improve treatment efficacy, and have lower costs, thus opening up new avenues for treatment. Although the potential of TCM and natural products in cancer therapy through ferroptosis has been confirmed, further in-depth studies are required to determine their effectiveness and safety, which will be the core focus of future research.

## Figures and Tables

**Table 1:** Ferroptosis-related proteins and molecular mechanisms.

Biological category		Genes name	Proteins name	Effect on ferroptotic types	Mode of action	Reference
Iron metabolism		DMT1	Divalent metal transporter1	Induces	Mediates the release of Fe^2+^ into a labile iron pool	7
		FPN	Ferroportin	Induces	Increasing iron-dependent lipid ROS accumulation	10
		FTH1	Ferritin heavy chain1	Induces	Iron accumulation Stores intracellular iron	9
		STEAP3	Six-transmembrane epithelial antigen of prostate3	Induces	Converts Fe^3+^ to Fe^2+^	12
		TfR1	Transferrin receptor1	Induces	Iron accumulation Imports iron into cells	6
Lipid metabolism		ACSL4	Acyl-CoA synthetase long-chain family member 4	Induces	Lipid ROS accumulation convertsAA/AdA intoAACoA/AdA CoA	17
		LOX	Lipoxygenases	Induces	Lipid peroxidation accumulation Generate lipid ROS by oxidizing AA-PE and AdA-PE	18
Glutaminolysis (glutamate uptake)		ASCT2 (SLC1A5)	Glutamine transporter	Inhibits	Glutamate/cystine transportation	28
		SLC38A1	Glutamine transporter	Inhibits	Glutamate/cystine transportation	28, 29
Amino acid metabolism (cystine uptake) GPX4-dependent antioxidant axis		GCL	Glutamate-cysteine ligase	Inhibits	Cysteine and glutamate to form the dipeptide gamma-glutamyl cysteine (γ-GC)	39
		GPX4	Glutathione peroxidase 4	Inhibits	Prevents lipids hydroperoxide formation	23
		GSR	Glutathione reductase	Inhibits	Catalyzes the reduction of GSSG to GSH using electrons provided by NADPH/H^+^	40
		SLC3A2	Solute carrier family 3 membrane 2	Inhibits	Maintains SLC7A11stability	22
		SLC7A11	Solute carrier family 7 membrane 11	Inhibits	Promotes cystine uptake	2
		TXNRD1	Thioredoxin reductase 1	Inhibits	Reduces thioredoxin and activates Trans-sulfuration pathway	25
GPX4-independent antioxidant axis						
Ⅰ.	FSP1/CoQ10/NAD(P)H axis	CoQ10	Coenzyme Q10	Inhibits	Mitochondrial oxidative phosphorylation	32
		FSP1	Ferroptosis suppressor protein-1	Inhibits	Parallel to the GSH-GPX4 pathway	33
Ⅱ.	Mevalonate pathway	HMG-CoA reductase	3-hydroxy-3-methylglutaryl-CoA reductase	Inhibits	GPX4 maturation	41
Ⅲ.	DHODH axis	DHODH	Dihydroorotate dehydrogenase	Inhibits	Coordinates with GPX4 to block ferroptosis in the mitochondrial inner membrane by reducing ubiquinone to ubiquinol	35
Ⅳ.	GCH1/BH4/DHFR/NAD(P)H axis	THB/BH4	Tetrahydrobiopterin	Inhibits	BH4 synthesis is a critical pathway involved in GPX4 inhibition	36
		DHFR	Dihydrofolate reductase	Inhibits	Reduces dihydrofolic acid to tetrahydrofolic acid	38
		GCH1	GTP Cyclohydrolase 1	Inhibits	Increases antioxidant BH4 and CoQ10 abundance	37

**Table 2:** Overview of the roles of ferroptosis-related biomarkers in GC.

Biological pathways	Biomarker genes	Biomarker proteins	Regulator of pro-/antiferroptotic proteins	Mode of action	Expression in GC tissue	Type of biomarker	Reference
Iron metabolism	TfR1	Transferrin receptor	Activator	Iron accumulation	High expression	Prognosis	42
Lipid metabolism	LOX15	Lipoxygenases15	Antiferroptotic proteins	●miR-522 target ●Depletes lipid ROS ●Chemoresistance	Low expression	Prognosis Chemoresistance	43
	CDO1	Cysteine dioxygenase1	Antiferroptotic proteins	Enhance antioxidant defense	Low expression	Prognosis	44
	PLIN2	Perilipin 2	Antiferroptotic proteins	Lipid ROS accumulation	High expression	Prognosis	45
	SCD1	Stearoyl-CoA desaturase1	Antiferroptotic proteins	Enhance antioxidant defense	High expression	Prognosis Development	46
xCT/GSH/GPX4-dependent antioxidant axis	GPX4	Glutathioneperoxidase4	Antiferroptotic proteins	Enhance antioxidant defense	High expression	Prognosis	47
	SLC7A11 (Xc)	Solute carrier family7 membrane11	Antiferroptotic proteins	Enhance antioxidant defense	High expression	Prognosis	48
GPX4-independent antioxidant axis	FSP1	Ferroptosis suppressor protein-1	Antiferroptotic proteins	Enhance antioxidant defense	High expression	Prognosis	49
Other metabolism	ATF2	Activating transcription factor 2	Pro-ferroptotic proteins	miR-496 target	High expression	Prognosis	50
	AURKA	Aurora Kinase A	Pro-ferroptotic proteins	miR-4715-3p target	High expression	Prognosis	51
	BAP31	B-cell receptor-associated protein 31	Antiferroptotic proteins	Interaction with Voltage-Dependent Anion Channel 1 (VDAC1), regulates ferroptosis.	High expression	Prognosis	52
	CDH1	E-cadherin	Pro-ferroptotic proteins	Activation of Hippo signaling pathway	Low expression	Prognosis	53
	CISD1	CDGSH iron sulfur domain 1	Antiferroptotic proteins	Regulation of mitochondrial iron metabolism	High expression	Prognosis	54
	DCLK3	Doublecortin-Like Kinase 3	Antiferroptotic proteins	Oxidative stress (mitochondria)	High expression	Prognosis	55
	GLS2	Glutaminase 2	Antiferroptotic proteins	miR-103a-3p target	Low expression	Development	56
	MGST1	Microsomal Glutathione S-Transferase 1	Antiferroptotic proteins	Enhancing the Wnt/β-Catenin Pathway	High expression	Prognosis	57
	NRF2	Nuclear factor-erythroid 2	Antiferroptotic proteins	●Reduce ROS accumulation ●Depletes lipid peroxidation	High expression	Prognosis	58
	PB	Polycyanin B	Pro-ferroptotic proteins	Regulate the expression of LC3B, TFR1, NOCA4, and FTH1	Low expression	Prognosis	59
	SLC1A5, ANGPTL4, CGAS	Ferroptosis-related genes/FRG signature	Pro-ferroptotic proteins	Erastin-induced	High expression	Prognosis	60
	STAT3	Signal transducer and activator of transcription 3	Pro-ferroptotic proteins	miR-125b-5p target	High expression	Prognosis	61
	ZFP36	Zinc finger protein 36 homolog	Pro-ferroptotic proteins	Erastin-induced	High expression	Prognosis	63

**Table 3:** Chemotherapeutic drug-related ferroptosis for GC.

Chemotherapy drugs used for GC treatment	Chemotherapy drug names	Pharmacological effects	Mode of action	Reference
Platinum-based	Cisplatin	Binds to DNA, affecting the synthesis of DNA, RNA, and protein	Silencing SIRT6 affects the Keap1/Nrf2 signaling pathway	64, 65
Platinum-based	Oxaliplatin	Thymidylate synthase	●miR-181a-5p may influence ferroptosis by regulating SIRT1 ●SIRT1-induced ferroptosis may be associated with GPX4 downregulation	66, 67
Paclitaxel-based	Sorafenib	Multi-tyrosine kinase inhibitors	●HSPH1 regulated by ATF2 plays a role in controlling ferroptosis. ●ATF3 inhibits the Nrf2/Keap1/xCT signaling pathway​	50
5-FU-based	Capecitabine	DNA synthesis inhibitor	ARF6 silencing enhances erastin-induced ferroptosis and lipid peroxidation	68
5-FU-based	5-FU	DNA synthesis inhibitor	Promotes ROS-mediated ferroptosis	69

**Table 4:** Ferroptosis-related Chinese herbal medicine/natural medicine/traditional Chinese medicine (TCM) for GC.

Class	Chinese herbal medicine/Natural medicine/ traditional Chinese medicine (TCM)	Active compound	Direct targets	Mode of action	Research model *in vitro* or *in vivo* (cell/animal)	Reference
Bioflavonoid	*Selaginella tamariscina*	Amentoflavone (AF)	miR-496/ATF2 axis	Increases ROS and lipid peroxidation, along with decreased GPX4 activity	AGS HGC-27	71
Biguanides	*Galega officinalis*	Metformin	STAT1	Disrupts the STAT1-PRMT1 axis	HGC-27 AGS	72, 73
Flavonoids	*Huang Qin* (*Scutellaria baicalensis*)	Baicalein	SLC7A11 p53 Fer-1	●p53-mediated SLC7A11 downregulation, leading to lipid peroxidation accumulation ●Baicalin-induced ferroptosis by reducing MDA levels and PTGS2 expression, improving cell survival	SGC-7901	74
Sesquiterpene lactone	*Artemisia annua* (*Artemisinin*)	Artesunate (ART) Dihydroartemisinin (DHA)	p53	Downregulates VEGF, upregulates calpain-2 expression	SGC-7901 BGC-823 MGC-803 AGS MKN74	84 75
TCM	*Actinidia Chinensis Planch* (*ACP*)	Ursolic acid (UA) Oleanolic acid (OA)	System Xc- GPX4 axis	Inhibits GPX4 and xCT expression, leading to ROS accumulation	HGC-27	76 77
TCM	*Yiqi Jiedu Huayu decoction* (*YJHD*)	ND	ACSL4 p53	Downregulates GPX4 and SLC7A11	HGC-27 Xenograft in zebrafish embryos	78, 79
Terpenes	*Maidong* *(Ophiopogon japonicus)*	Ophiopogonin B (OP-B)	GPX4 SLC7A11	Inhibits GPX4/Xc-system, leading to lipid ROS accumulation and reduced lipid ROS scavenging	AGS NCI-N87 Xenograft in mouse model	80
Terpenes	*Jiyuan Donglingcao* *(Rabdosia rubescens)*	Compound a2/ Jiyuan oridonin A (JDA)	GPX4	Decreased GPX4 induced the accumulation of Fe2^+^	MGC-803 MKN-45 Xenograft in mouse model	81
Quinones	*Danshen* *(Salvia miltiorrhiza Bunge)*	Tanshinone ⅡA (Tan ⅡA) Dihydroisotanshinone I (DT)	p53	Upregulates p53, decreases GSH and L-cysteine levels	BGC-823 NCI-N87 Xenograft in NOD SCID mice	82
			SLC7A11	Downregulates SLC7A11 and GPX4, GCSC stemness markers (OCT3/4, ALDH1A1)	GC stem cells (GCSC)	83

## Abbreviations

α-KG: α-ketoglutarate
α-TOH: α-tocopherol
γ-GC: gamma-glutamyl cysteine
5-FU: 5-fluoruracil
ACP: Actinidia chinensis Planch
ACSL4: acyl-coenzyme A synthetase long-chain family member 4
AF: Amentoflavone
ALOX: arachidonate lipoxygenase
ALDH1A1: Aldehyde dehydrogenase 1 family member A1
AURKA: Aurora kinase A
*Artemisinin*: *Artemisia annua*
ARF6: ADP-ribosylation factor 6
ART: Artesunate
ATF2: activating transcription factor 2
BAP31: B-cell receptor-associated protein 31
CBS: cystathionine β-synthase
CDH1: E-cadherin
CDO1: cysteine dioxygenase 1
GC: gastric cancer
CISD1: CDGSH iron sulfur domain 1
CTH: cystathionine gamma-lyase
CoQ10: coenzyme Q10
DGC: diffuse-type gastric cancer
DHA: Dihydroartemisinin
DCLK3: doublecortin-like kinase 3
DHA: Dihydroartemisinin
DHFR: Dihydrofolate reductase
DHODH: Dihydroorotate dehydrogenase
DMT1: Divalent metal transporter1
DT: Dihydroisotanshinone I
EMT: epithelial-mesenchymal transition
Fe²⁺: ferrous iron
Fe³⁺: ferric iron
FE: ferritin
FPN: ferroportin
FSP1: Ferroptosis suppressor protein 1
FRG: ferroptosis-related genes signature
FTH1: Ferritin heavy chain1
GCH1: GTP cyclohydrolase1
GC: gastric cancer
GCL: Glutamate-cysteine ligase
Gln: glutamate
GLS: glutaminase
GNMT: glycine N-methyltransferase
GPX4: glutathione peroxidase 4
GSH: glutathione
GSR: Glutathione reductase
GSSG: glutathione disulfide
HMG-CoA reductase: 3-hydroxy-3-methylglutaryl-CoA reductase
HSPH1/HSPH105/HSPH110: heat shock protein 110
JDA: Jiyuan oridonin A
LPCAT3: lysophosphatidylcholine acyltransferase 3
LOX15: lipoxygenases 15
MGST1: microsomal glutathione S-Transferase 1
MVA: Mevalonate
NAD(P)H: nicotinamide adenine dinucleotide phosphate
NRF2: nuclear factor-erythroid 2
OA: oleanolic acid
OCT3/4: Octamer binding transcription factor 3/4
OP-B: Ophiopogonin B
PB: polycyanin B
PLOOH: phospholipid hydroperoxide
PLIN2: perilipin 2
PUFAs: polyunsaturated fatty acids
RCD: regulated cell death
ROS: reactive oxygen species
SCD1: stearoyl-CoA desaturase 1
SAHH: S-adenosylhomocysteine hydrolase
SIRT6: sirtuin 6
SLC3A2: solute carrier family 3 member 2
SLC7A11: solute carrier family 7 member 11
STAT3: signal transducer activator of transcription 3
STEAP3: Six-transmembrane epithelial antigen of prostate3
Tan IIA: Tanshinone IIA
TCA: tricarboxylic acid
TCM: traditional Chinese medicines
TfR1: transferrin receptor 1
THB: tetrahydrobiopterin
TXN: thioredoxin
TXNRD1: thioredoxin reductase 1
UA: ursolic acid
VDAC1: Voltage-Dependent Anion Channel 1
YJHD: *Yiqi Jiedu Huayu* decoction
ZFP36: zinc finger protein 36 homolog

## References

[1] Dolma S, Lessnick SL, Hahn WC, Stockwell BR (2003). Identification of genotype-selective antitumor agents using synthetic lethal chemical screening in engineered human tumor cells. Cancer Cell.

[2] Dixon SJ, Lemberg KM, Lamprecht MR, Skouta R, Zaitsev EM, Gleason CE (2012). Ferroptosis: an iron-dependent form of nonapoptotic cell death. Cell.

[3] Venkataramani V (2021). Iron Homeostasis and Metabolism: Two Sides of a Coin. Adv Exp Med Biol.

[4] Li B, Cheng K, Wang T, Peng X, Xu P, Liu G (2024). Research progress on GPX4 targeted compounds. Eur J Med Chem.

[5] Evstatiev R, Gasche C (2012). Iron sensing and signalling. Gut.

[6] Gammella E, Buratti P, Cairo G, Recalcati S (2017). The transferrin receptor: the cellular iron gate. Metallomics.

[7] Galy B, Ferring-Appel D, Becker C, Gretz N, Grone HJ, Schumann K (2013). Iron regulatory proteins control a mucosal block to intestinal iron absorption. Cell Rep.

[8] Enko D (2025). Physiology of Iron Metabolism. Clin Lab.

[9] Shpyleva SI, Tryndyak VP, Kovalchuk O, Starlard-Davenport A, Chekhun VF, Beland FA (2011). Role of ferritin alterations in human breast cancer cells. Breast Cancer Res Treat.

[10] Vlasveld LT, Janssen R, Bardou-Jacquet E, Venselaar H, Hamdi-Roze H, Drakesmith H (2019). Twenty Years of Ferroportin Disease: A Review or An Update of Published Clinical, Biochemical, Molecular, and Functional Features. Pharmaceuticals (Basel).

[11] Guggisberg CA, Kim J, Lee J, Chen X, Ryu MS (2022). NCOA4 Regulates Iron Recycling and Responds to Hepcidin Activity and Lipopolysaccharide in Macrophages. Antioxidants (Basel).

[12] Song X, Xie Y, Kang R, Hou W, Sun X, Epperly MW (2016). FANCD2 protects against bone marrow injury from ferroptosis. Biochem Biophys Res Commun.

[13] Liu K, Huang L, Qi S, Liu S, Xie W, Du L (2023). Ferroptosis: The Entanglement between Traditional Drugs and Nanodrugs in Tumor Therapy. Adv Healthc Mater.

[14] Gu R, Xia Y, Li P, Zou D, Lu K, Ren L (2022). Ferroptosis and its Role in Gastric Cancer. Front Cell Dev Biol.

[15] Su Y, Zhao B, Zhou L, Zhang Z, Shen Y, Lv H (2020). Ferroptosis, a novel pharmacological mechanism of anti-cancer drugs. Cancer Lett.

[16] Wang M, Thanou M (2010). Targeting nanoparticles to cancer. Pharmacol Res.

[17] Doll S, Proneth B, Tyurina YY, Panzilius E, Kobayashi S, Ingold I (2017). ACSL4 dictates ferroptosis sensitivity by shaping cellular lipid composition. Nat Chem Biol.

[18] Seiler A, Schneider M, Forster H, Roth S, Wirth EK, Culmsee C (2008). Glutathione peroxidase 4 senses and translates oxidative stress into 12/15-lipoxygenase dependent- and AIF-mediated cell death. Cell Metab.

[19] Kim SW, Kim Y, Kim SE, An JY (2021). Ferroptosis-Related Genes in Neurodevelopment and Central Nervous System. Biology (Basel).

[20] Prabhune NM, Ameen B, Prabhu S (2025). Therapeutic potential of synthetic and natural iron chelators against ferroptosis. Naunyn Schmiedebergs Arch Pharmacol.

[21] Bai C, Hua J, Meng D, Xu Y, Zhong B, Liu M Glutaminase-1 Mediated Glutaminolysis to Glutathione Synthesis Maintains Redox Homeostasis and Modulates Ferroptosis Sensitivity in Cancer Cells. Cell Prolif. 2025: e70036.

[22] Koppula P, Zhang Y, Zhuang L, Gan B (2018). Amino acid transporter SLC7A11/xCT at the crossroads of regulating redox homeostasis and nutrient dependency of cancer. Cancer Commun (Lond).

[23] Zou Y, Palte MJ, Deik AA, Li H, Eaton JK, Wang W (2019). A GPX4-dependent cancer cell state underlies the clear-cell morphology and confers sensitivity to ferroptosis. Nat Commun.

[24] Arner ESJ, Schmidt EE (2024). Unresolved questions regarding cellular cysteine sources and their possible relationships to ferroptosis. Adv Cancer Res.

[25] Hsieh MS, Ling HH, Setiawan SA, Hardianti MS, Fong IH, Yeh CT (2024). Therapeutic targeting of thioredoxin reductase 1 causes ferroptosis while potentiating anti-PD-1 efficacy in head and neck cancer. Chem Biol Interact.

[26] Miller CG, Holmgren A, Arner ESJ, Schmidt EE (2018). NADPH-dependent and -independent disulfide reductase systems. Free Radic Biol Med.

[27] Chen NC, Yang F, Capecci LM, Gu Z, Schafer AI, Durante W (2010). Regulation of homocysteine metabolism and methylation in human and mouse tissues. FASEB J.

[28] Gao M, Monian P, Quadri N, Ramasamy R, Jiang X (2015). Glutaminolysis and Transferrin Regulate Ferroptosis. Mol Cell.

[29] Xu J, Bai X, Dong K, Du Q, Ma P, Zhang Z (2025). GluOC Induced SLC7A11 and SLC38A1 to Activate Redox Processes and Resist Ferroptosis in TNBC. Cancers (Basel).

[30] He R, Wei Y, Peng Z, Yang J, Zhou Z, Li A (2024). alpha-Ketoglutarate alleviates osteoarthritis by inhibiting ferroptosis via the ETV4/SLC7A11/GPX4 signaling pathway. Cell Mol Biol Lett.

[31] Fujii J, Yamada KI (2023). Defense systems to avoid ferroptosis caused by lipid peroxidation-mediated membrane damage. Free Radic Res.

[32] Santoro MM (2020). The Antioxidant Role of Non-mitochondrial CoQ10: Mystery Solved!. Cell Metab.

[33] Doll S, Freitas FP, Shah R, Aldrovandi M, da Silva MC, Ingold I (2019). FSP1 is a glutathione-independent ferroptosis suppressor. Nature.

[34] Sun Q, Liu D, Cui W, Cheng H, Huang L, Zhang R (2023). Cholesterol mediated ferroptosis suppression reveals essential roles of Coenzyme Q and squalene. Commun Biol.

[35] Amos A, Amos A, Wu L, Xia H (2023). The Warburg effect modulates DHODH role in ferroptosis: a review. Cell Commun Signal.

[36] Vasquez-Vivar J, Shi Z, Tan S (2022). Tetrahydrobiopterin in Cell Function and Death Mechanisms. Antioxid Redox Signal.

[37] Liu M, Kong XY, Yao Y, Wang XA, Yang W, Wu H (2022). The critical role and molecular mechanisms of ferroptosis in antioxidant systems: a narrative review. Ann Transl Med.

[38] Zheng J, Conrad M (2025). Ferroptosis: when metabolism meets cell death. Physiol Rev.

[39] Wu G, Fang YZ, Yang S, Lupton JR, Turner ND (2004). Glutathione metabolism and its implications for health. J Nutr.

[40] Wu W, Geng Z, Bai H, Liu T, Zhang B (2021). Ammonium Ferric Citrate induced Ferroptosis in Non-Small-Cell Lung Carcinoma through the inhibition of GPX4-GSS/GSR-GGT axis activity. Int J Med Sci.

[41] Yin Z, Shen G, Fan M, Zheng P (2025). Lipid metabolic reprogramming and associated ferroptosis in osteosarcoma: From molecular mechanisms to potential targets. J Bone Oncol.

[42] Cheng X, Fan K, Wang L, Ying X, Sanders AJ, Guo T (2020). TfR1 binding with H-ferritin nanocarrier achieves prognostic diagnosis and enhances the therapeutic efficacy in clinical gastric cancer. Cell Death Dis.

[43] Zhang H, Deng T, Liu R, Ning T, Yang H, Liu D (2020). CAF secreted miR-522 suppresses ferroptosis and promotes acquired chemo-resistance in gastric cancer. Mol Cancer.

[44] Jiang X, Yan Q, Xie L, Xu S, Jiang K, Huang J (2021). Construction and Validation of a Ferroptosis-Related Prognostic Model for Gastric Cancer. J Oncol.

[45] Sun X, Yang S, Feng X, Zheng Y, Zhou J, Wang H (2020). The modification of ferroptosis and abnormal lipometabolism through overexpression and knockdown of potential prognostic biomarker perilipin2 in gastric carcinoma. Gastric Cancer.

[46] Sen U, Coleman C, Sen T (2023). Stearoyl coenzyme A desaturase-1: multitasker in cancer, metabolism, and ferroptosis. Trends Cancer.

[47] Sugezawa K, Morimoto M, Yamamoto M, Matsumi Y, Nakayama Y, Hara K (2022). GPX4 Regulates Tumor Cell Proliferation via Suppressing Ferroptosis and Exhibits Prognostic Significance in Gastric Cancer. Anticancer Res.

[48] Lin Y, Dong Y, Liu W, Fan X, Sun Y (2022). Pan-Cancer Analyses Confirmed the Ferroptosis-Related Gene SLC7A11 as a Prognostic Biomarker for Cancer. Int J Gen Med.

[49] Tamura K, Tomita Y, Kanazawa T, Shinohara H, Sakano M, Ishibashi S (2024). Lipid Peroxidation Regulators GPX4 and FSP1 as Prognostic Markers and Therapeutic Targets in Advanced Gastric Cancer. Int J Mol Sci.

[50] Xu X, Li Y, Wu Y, Wang M, Lu Y, Fang Z (2023). Increased ATF2 expression predicts poor prognosis and inhibits sorafenib-induced ferroptosis in gastric cancer. Redox Biol.

[51] Gomaa A, Peng D, Chen Z, Soutto M, Abouelezz K, Corvalan A (2019). Epigenetic regulation of AURKA by miR-4715-3p in upper gastrointestinal cancers. Sci Rep.

[52] Lin L, Que R, Wang J, Zhu Y, Liu X, Xu R (2022). Prognostic value of the ferroptosis-related gene SLC2A3 in gastric cancer and related immune mechanisms. Front Genet.

[53] Minikes AM, Song Y, Feng Y, Yoon C, Yoon SS, Jiang X (2023). E-cadherin is a biomarker for ferroptosis sensitivity in diffuse gastric cancer. Oncogene.

[54] Zang J, Cui M, Xiao L, Zhang J, Jing R (2023). Overexpression of ferroptosis-related genes FSP1 and CISD1 is related to prognosis and tumor immune infiltration in gastric cancer. Clin Transl Oncol.

[55] Cheng J, Tang YC, Dong Y, Qin RL, Dong ZQ (2024). Doublecortin-like kinase 3 (DCLK3) is associated with bad clinical outcome of patients with gastric cancer and regulates the ferroptosis and mitochondria function in vitro and in vivo. Ir J Med Sci.

[56] Li D, Cao D, Zhang Y, Yu X, Wu Y, Jia Z (2025). Integrative pan-cancer analysis and experiment validation identified GLS as a biomarker in tumor progression, prognosis, immune microenvironment, and immunotherapy. Sci Rep.

[57] Li Y, Xu X, Wang X, Zhang C, Hu A, Li Y (2023). MGST1 Expression Is Associated with Poor Prognosis, Enhancing the Wnt/beta-Catenin Pathway via Regulating AKT and Inhibiting Ferroptosis in Gastric Cancer. ACS Omega.

[58] Li X, Qian J, Xu J, Bai H, Yang J, Chen L (2024). NRF2 inhibits RSL3 induced ferroptosis in gastric cancer through regulation of AKR1B1. Exp Cell Res.

[59] Hu C, Zu D, Xu J, Xu H, Yuan L, Chen J (2023). Polyphyllin B Suppresses Gastric Tumor Growth by Modulating Iron Metabolism and Inducing Ferroptosis. Int J Biol Sci.

[60] Wang F, Chen C, Chen WP, Li ZL, Cheng H (2021). Development and Validation of a Novel Ferroptosis-Related Gene Signature for Predicting Prognosis and the Immune Microenvironment in Gastric Cancer. Biomed Res Int.

[61] Liu YP, Qiu ZZ, Li XH, Li EY (2021). Propofol induces ferroptosis and inhibits malignant phenotypes of gastric cancer cells by regulating miR-125b-5p/STAT3 axis. World J Gastrointest Oncol.

[62] Expression of concern (2022). Ophiopogonin B induces the autophagy and apoptosis of colon cancer cells by activating JNK/c-Jun signaling pathway [Biomedicine & Pharmacotherapy 108 (2018) 1208-1215]. Biomed Pharmacother.

[63] Zhang S, Li Z, Hu G, Chen H (2025). Integrative single-cell and multi-omics analyses reveal ferroptosis-associated gene expression and immune microenvironment heterogeneity in gastric cancer. Discov Oncol.

[64] Feng S, Li Y, Huang H, Huang H, Duan Y, Yuan Z (2023). Isoorientin reverses lung cancer drug resistance by promoting ferroptosis via the SIRT6/Nrf2/GPX4 signaling pathway. Eur J Pharmacol.

[65] Fu D, Wang C, Yu L, Yu R (2021). Induction of ferroptosis by ATF3 elevation alleviates cisplatin resistance in gastric cancer by restraining Nrf2/Keap1/xCT signaling. Cell Mol Biol Lett.

[66] Zhong S, Wang Z, Yang J, Jiang D, Wang K (2024). Ferroptosis-related oxaliplatin resistance in multiple cancers: Potential roles and therapeutic Implications. Heliyon.

[67] Qu X, Liu B, Wang L, Liu L, Zhao W, Liu C (2023). Loss of cancer-associated fibroblast-derived exosomal DACT3-AS1 promotes malignant transformation and ferroptosis-mediated oxaliplatin resistance in gastric cancer. Drug Resist Updat.

[68] Geng D, Wu H (2022). Abrogation of ARF6 in promoting erastin-induced ferroptosis and mitigating capecitabine resistance in gastric cancer cells. J Gastrointest Oncol.

[69] Yuan J, Khan SU, Yan J, Lu J, Yang C, Tong Q (2023). Baicalin enhances the efficacy of 5-Fluorouracil in gastric cancer by promoting ROS-mediated ferroptosis. Biomed Pharmacother.

[70] Galluzzi L, Vitale I, Aaronson SA, Abrams JM, Adam D, Agostinis P (2018). Molecular mechanisms of cell death: recommendations of the Nomenclature Committee on Cell Death 2018. Cell Death Differ.

[71] Tang F, Xu Y, Gao E, Zhang W, Zhang F, Xiang Y (2023). Amentoflavone attenuates cell proliferation and induces ferroptosis in human gastric cancer by miR-496/ATF2 axis. Chem Biol Drug Des.

[72] Sun S, Shen J, Jiang J, Wang F, Min J (2023). Targeting ferroptosis opens new avenues for the development of novel therapeutics. Signal Transduct Target Ther.

[73] Wang K, Chen Y, Zhang M, Wang S, Yao S, Gong Z (2024). Metformin suppresses gastric cancer progression by disrupting the STAT1-PRMT1 axis. Biochem Pharmacol.

[74] Shao L, Zhu L, Su R, Yang C, Gao X, Xu Y (2024). Baicalin enhances the chemotherapy sensitivity of oxaliplatin-resistant gastric cancer cells by activating p53-mediated ferroptosis. Sci Rep.

[75] Zhang Y, Xu G, Zhang S, Wang D, Saravana Prabha P, Zuo Z (2018). Antitumor Research on Artemisinin and Its Bioactive Derivatives. Nat Prod Bioprospect.

[76] Gao Z, Deng G, Li Y, Huang H, Sun X, Shi H (2020). Actinidia chinensis Planch prevents proliferation and migration of gastric cancer associated with apoptosis, ferroptosis activation and mesenchymal phenotype suppression. Biomed Pharmacother.

[77] Lee JY, Nam M, Son HY, Hyun K, Jang SY, Kim JW (2020). Polyunsaturated fatty acid biosynthesis pathway determines ferroptosis sensitivity in gastric cancer. Proc Natl Acad Sci U S A.

[78] Xi S, Ding W, Weng D, Zeng Y, Gao K, Wu Q (2024). Chrysophanol induces apoptosis and ferroptosis of gastric cancer cells by targeted regulation of mTOR. Chem Biol Drug Des.

[79] Song S, Wen F, Gu S, Gu P, Huang W, Ruan S (2022). Network Pharmacology Study and Experimental Validation of Yiqi Huayu Decoction Inducing Ferroptosis in Gastric Cancer. Front Oncol.

[80] Zhang L, Li C, Zhang Y, Zhang J, Yang X (2022). Ophiopogonin B induces gastric cancer cell death by blocking the GPX4/xCT-dependent ferroptosis pathway. Oncol Lett.

[81] Liu Y, Song Z, Liu Y, Ma X, Wang W, Ke Y (2021). Identification of ferroptosis as a novel mechanism for antitumor activity of natural product derivative a2 in gastric cancer. Acta Pharm Sin B.

[82] Guan Z, Chen J, Li X, Dong N (2020). Tanshinone IIA induces ferroptosis in gastric cancer cells through p53-mediated SLC7A11 down-regulation. Biosci Rep.

[83] Ni H, Ruan G, Sun C, Yang X, Miao Z, Li J (2022). Tanshinone IIA inhibits gastric cancer cell stemness through inducing ferroptosis. Environ Toxicol.

[84] Hu Y, Guo N, Yang T, Yan J, Wang W, Li X (2022). The Potential Mechanisms by which Artemisinin and Its Derivatives Induce Ferroptosis in the Treatment of Cancer. Oxid Med Cell Longev.

